# Factors influencing the rollout and uptake of COVID-19 rapid diagnostic testing: qualitative insights from six African nations

**DOI:** 10.3389/fpubh.2025.1551907

**Published:** 2025-10-15

**Authors:** Patrick A. Njukeng, Charles Njumkeng, Prudence Tatiana Nti Mvilongo, Elvis T. Amin, Akemfua Fualefac, Serge Raoul Ekukole Ekome, Abderahim Mahamat Nadjib, Lethso Thibaut Ocko Gokaba, Ousmane Sani, Bandaogo Ousséni, Alusine Fofana, Kah Evans Ngha, Kekeletso Kao

**Affiliations:** ^1^Global Health Systems Solutions, Douala, Cameroon; ^2^Department of Microbiology and Parasitology, University of Buea, Buea, Cameroon; ^3^Department of Public Health and Hygiene, University of Buea, Buea, Cameroon; ^4^Faculté des Sciences de la Santé Humaine, Université de N'Djamena, N'Djamena, Chad; ^5^Faculté des Sciences de la Santé, Université Marien Ngouabi, Brazzaville, Republic of Congo; ^6^Centre de Recherche Médicale et Sanitaire, Niamey, Niger; ^7^Institut National de Santé Publique, Ouagadougou, Burkina Faso; ^8^Ministry of Health and Sanitation, Freetown, Sierra Leone; ^9^Department of Anthropology, University of Yaoundé I, Yaoundé, Cameroon; ^10^Foundation for Innovative New Diagnostics, Geneva, Switzerland

**Keywords:** COVID-19, rapid diagnostic tests, rollout, uptake, Africa, community engagement

## Abstract

**Introduction:**

The COVID-19 pandemic has profoundly affected healthcare systems in Africa, revealing urgent Challenges in resource-limited settings and the need for effective diagnostic strategies. This study examines the factors influencing the rollout and uptake of COVID-19 antigen-based rapid diagnostic tests (Ag-RDTs) in six African countries: Cameroon, Chad, Burkina Faso, Niger, Sierra Leone, and the Republic of Congo.

**Methods:**

Utilizing qualitative methodologies, we conducted focus group discussions (FGDs) with healthcare personnel, community health workers, and community members to explore barriers and facilitators affecting decentralized testing efforts.

**Results:**

Key barriers identified include misinformation, distrust in government intentions, staff demotivation, and stigma surrounding positive test results. In contrast, facilitators such as community engagement, short turn around turn, international travel requirement and the involvement of community health workers in testing process emerged as strong motivators for testing uptake. Findings emphasize the critical importance of coherent communication strategies and community sensitization efforts to combat misinformation and foster acceptance of testing. Moreover, Integrating COVID-19 testing into routine healthcare surveillance and strengthening community health systems through capacity building are essential for improving overall public health responses. Stakeholders highlighted government policies, including public awareness campaigns, Media engagement and decentralisation of testing services, as essential in improving testing coverage.

**Conclusion:**

This study underscores the need for targeted interventions that respect local contexts, enhancing the resilience and strength of healthcare systems and pandemic preparedness in Africa against current and future public health challenges.

## Introduction

The Coronavirus disease 2019 (COVID-19) pandemic has profoundly altered daily life across the globe, significantly impacting healthcare systems, particularly in resource-constrained regions such as Africa (1). As of February 2023, Africa recorded over 10.8 million COVID-19 cases and 228,738 deaths, compounding the strain on the continent’s already fragile healthcare structure (2, 3). Meanwhile, new variants led to a surge in infections, complicating public health responses (4, 5). This highlighted an urgent need for effective testing strategies; however, molecular testing capacity was insufficient to meet demand (6). In resource-limited settings, barriers to testing included a shortage of trained personnel, inadequate infrastructure, and long turnaround times for results (6).

In response, the World Health Organization (WHO) recommended the use of antigen-based rapid diagnostic tests (Ag-RDTs), emphasizing decentralized testing approaches that can be implemented at or near points of care (7–9). To further enhance COVID-19 screening, the WHO launched an initiative aimed at reaching more than 7 million individuals through community testing initiatives, targeting a 40% testing coverage (10, 11). Concurrently, the Africa Centers for Disease Control and Prevention (Africa CDC) initiated the Partnership to Accelerate COVID-19 Testing (PACT), which sought to amplify testing efforts across the continent and mitigate the transmission of the virus (10). WHO’s endorsement of Ag-RDTs has been pivotal, facilitating widespread testing to enable early identification and curtail transmission (12).

While the global response to the COVID-19 pandemic has underscored the critical need for accessible diagnostics, existing literature often broadly addresses testing challenges in resource-limited settings without specifically detailing the nuanced experiences of key populations involved in RDT rollout and uptake (10, 13). Research on the distinct perceptions, motivations, and barriers encountered by healthcare personnel, community health workers, and community members in African contexts regarding Ag-RDTs remains limited (3, 14). Understanding these specific perspectives is crucial for developing effective and contextually relevant strategies to enhance testing coverage and preparedness for future health crises.

In a collaborative effort, Global Health Systems Solutions (GHSS), a non-governmental organization based in Cameroon, received funding from the Foundation for Innovative New Diagnostics (FIND) to enhance COVID-19 responses and escalate rapid testing across six African countries: Cameroon, the Republic of Congo, Chad, Niger, Burkina Faso, and Sierra Leone. This initiative spanned from December 2021 to January 2023 aiming to refine rapid diagnostic testing strategies, build capacity, ensure quality assurance for test kits, and enhance community surveillance (10). Despite these initiatives, a significant gap remained in the accessibility and uptake of COVID-19 testing services. Reports indicate that only 14.2% of COVID-19 infections are detected, primarily among symptomatic individuals and international travelers (15–17). This under-reporting underscored the critical need to innovate testing strategies, particularly given the high prevalence of asymptomatic cases (12, 18).

As African nations progress towards pandemic remission, they are strengthening their healthcare systems by integrating COVID-19 testing into routine respiratory disease surveillance (2). Reliable testing is crucial for effective pandemic preparedness, significantly affecting patient care and public health interventions (19). To inform and facilitate sustained and contextualized integration, we conducted a qualitative research to identify the barriers and facilitators affecting the rollout and uptake of rapid diagnostic COVID-19 testing in community surveillance across six African member states, aimed at preventing resurgence and improving preparedness.

## Materials and methods

This qualitative study was conducted at selected sites in six African countries—Burkina Faso, Cameroon, Chad, Congo-Brazzaville, Niger, and Sierra Leone (see Supplementary material 1). These countries were strategically chosen as part of a larger initiative aimed at enhancing COVID-19 response efforts in Africa, with a focus on their low testing rates. Despite these efforts, persistent under-reporting of COVID-19 underscored the urgent need to understand the factors influencing testing uptake within these diverse contexts. Including countries that span different regions, health systems, and public health environments was essential for exploring the challenges and facilitators of implementing decentralized rapid testing.

To achieve this, we conducted Focus Group Discussions (FGDs) to explore barriers and facilitators related to the rollout and uptake of COVID-19 rapid diagnostic testing (RDT). The findings of this study are reported in accordance with the COREQ (Consolidated Criteria for Reporting Qualitative Research) checklist (see Supplementary file 3).

### Research team and reflexivity

Six authors were involved in conducting the FGDs across the six countries this included; AF, MT, CO, CN, ETA, AA; Table 1 summarizes the researcher’s credentials, their occupation, gender and the country where they conducted the FGDs. All FGD moderators participated in a briefing session on two discussion guides—one for healthcare providers and one for community members which were pilot tested. The Flexible semi-structured interview guides included open-ended questions and prompts covering topics such as knowledge and perceptions of COVID-19, testing uptake, motivators and barriers, misinformation, and strategies to enhance testing at the individual, community, and health system levels (see Supplementary file 4 for the guides). The lead moderators had no prior interaction with participants before the FGD and explained the research objectives, procedures, and the intended dissemination of findings, which aim to inform policy and practice for future pandemic surveillance. To minimize potential influence, external moderators were engaged per country. Participants were reassured that their perceptions would be used solely for research purposes and would not affect their care.

**Table 1:** Characteristics of main moderators for the FGDs.

Moderators	Gender	Credentials/area of expertise	Coordinated FGD
AF	M	- BMLS (Bachelor of medical laboratory Science)/MSc (Master in Microbiology) - Quality assurance officer	Burkina Faso, Congo Brazzaville, Sierra Leone,
MT	F	- Doctor of Medicine (MD)/Masters in Public Health (MPH) - Health Systems and Disease Control - Public health Officer	
CO	M	- BNS (Bachelor of Nursing Science)/ Masters in Public Health (MPH) - Quality Assurance officer	
CN	M	- BMLS (Bachelor of medical laboratory Science)/MSc in Epidemiology - Lab System Strengthening / Epidemiologist	Niger, Chad, Cameroon
ETA	M	- Doctor of Medicine (MD)/Masters in Public Health (MPH) - Health Systems and Disease Control - Public health Officer	
AA	F	- Bsc in Microbiology/Msc in Microbiology - Quality assurance officer	

### Study design

The research employed a qualitative design guided by thematic analysis, a flexible and interpretive approach that permitted us to focuses on identify, analyze, and reporting themes within the related factors influencing the rollout and uptake of COVID-19 rapid diagnostic testing within the context of the six study countries. The FGDs included community members and healthcare providers, with key informants identified through purposive sampling from November 22 to February 2023, using maximum variation sampling based on gender, age, and roles in the COVID-19 rollout process. Each FDG organized, consisted of 6–12 participants and lasted 45 to 60 min. Participation was voluntary, and written informed consent was obtained from all participants before enrollment in the study. Interviews were conducted in English and French and were audio recorded. The duration and number of FGDs were designed to achieve data saturation.

The recruitment of health workers was purposive, targeting providers actively involved in COVID-19 case detection, testing, and vaccination. This included medical doctors, nurses, laboratory personnel, public health officers, and community health workers (see Table 2 for participant characteristics). Researchers approached these providers face-to-face at selected study sites and invited them to participate voluntarily (refer to Supplementary file 1 for the supported sites). For community members, convenience sampling was used to recruit participants face-to-face during routine hospital visits or outreach activities. Eligible participants were aged 18 and above, able to provide informed consent, and willing to participate voluntarily regardless of whether they had been tested for COVID-19, in order to gather their perceptions on COVID-19 testing uptake. Given the large scale of the study, individuals who did not meet the inclusion criteria or declined to participate were not included. Although this data was not formally documented, the primary reason for non-participation was their reluctance to be associated with COVID-19-related discussions, which they perceived as politically sensitive.

**Table 2:** Characteristics of study participant.

Country	Category	Sessions	Participants	Gender		Age group				Position (if Health Worker)
				Male	Female	18–25	26–35	36–45	> 45	
Congo										
	Health workers	3	33	18	15	9	10	12	2	8 Lab Scientists, 8 Nurses, 3 Pharmacists, 3 Medical Doctors, 11 CHW
	Community	3	32	12	20	7	9	10	6	
	Sub-total	6	65	30	35	16	19	22	8	
Burkina Faso										
	Health workers	5	50	27	23	6	19	18	7	5 Lab Scientists, 5 Clinical Biologists, 10Public Health Officers, 5 Medical Doctor, 5 Nurses, 20CHWs,
	Community	5	50	26	24	6	21	17	6	
	Sub-total	10	100	53	47	12	40	35	13	
Sierra Leone										
	Health workers	3	33	17	16	6	16	7	4	6 Lab scientists, 3 Lab Technicians, 3 Public Health Officer, 3 Medical Doctors, 6 Nurses, 12 CHWs
	Community	3	30	18	12	1	14	11	4	
	Sub-total	6	63	35	28	7	30	18	8	
Chad										
	Health workers	3	30	17	13	8	7	10	5	3 Lab Scientists, 6 Lab technicians, 3 Nurses, 3 Medical Doctors, 15 CHWs
	Community	3	25	15	10	4	14	6	1	
	Sub-total	6	55	32	23	12	21	16	6	
Niger										
	Health workers	4	45	17	28	7	12	16	10	8 Lab technicians, 8 Public Health Officer, 4 Medical doctor, 8 Nurses, 4 Pharmacist, 13 CHWs
	Community	4	40	10	30	6	12	13	9	
	Sub-total	8	85	27	58	13	24	29	19	
Cameroon										
	Health workers	1	10	4	6	0	6	4	0	4 CHWs, 1 Nurse, 1 Medical Doctor, 2 Lab Scientists, 1 pharmacist, 1 public health Officer
	Community	1	10	5	5	0	8	2	0	
	Sub-total	2	20	9	11	0	14	6	0	
Grand Total		36	388	186	202	60	148	126	54	
(%)				48%	52%	16%	38%	32%	14%	

### Data management

Audio recordings from the FGD were transcribed locally by research teams, with transcripts in French translated into English by the Juryman translation agency. To ensure accuracy and data integrity, bilingual local moderators reviewed and validated the translations in consultation with selected FGD participants. The transcripts were then anonymized to remove all direct identifiers and reviewed for quality to maintain participant confidentiality. Pseudonymized data was securely stored on a password-protected GHSS OneDrive on Teams, accessible only to authorized research team members. Once transcription verification was completed by the Principal Investigator, the original audio recordings were deleted.

### Analysis

**Figure 1:**
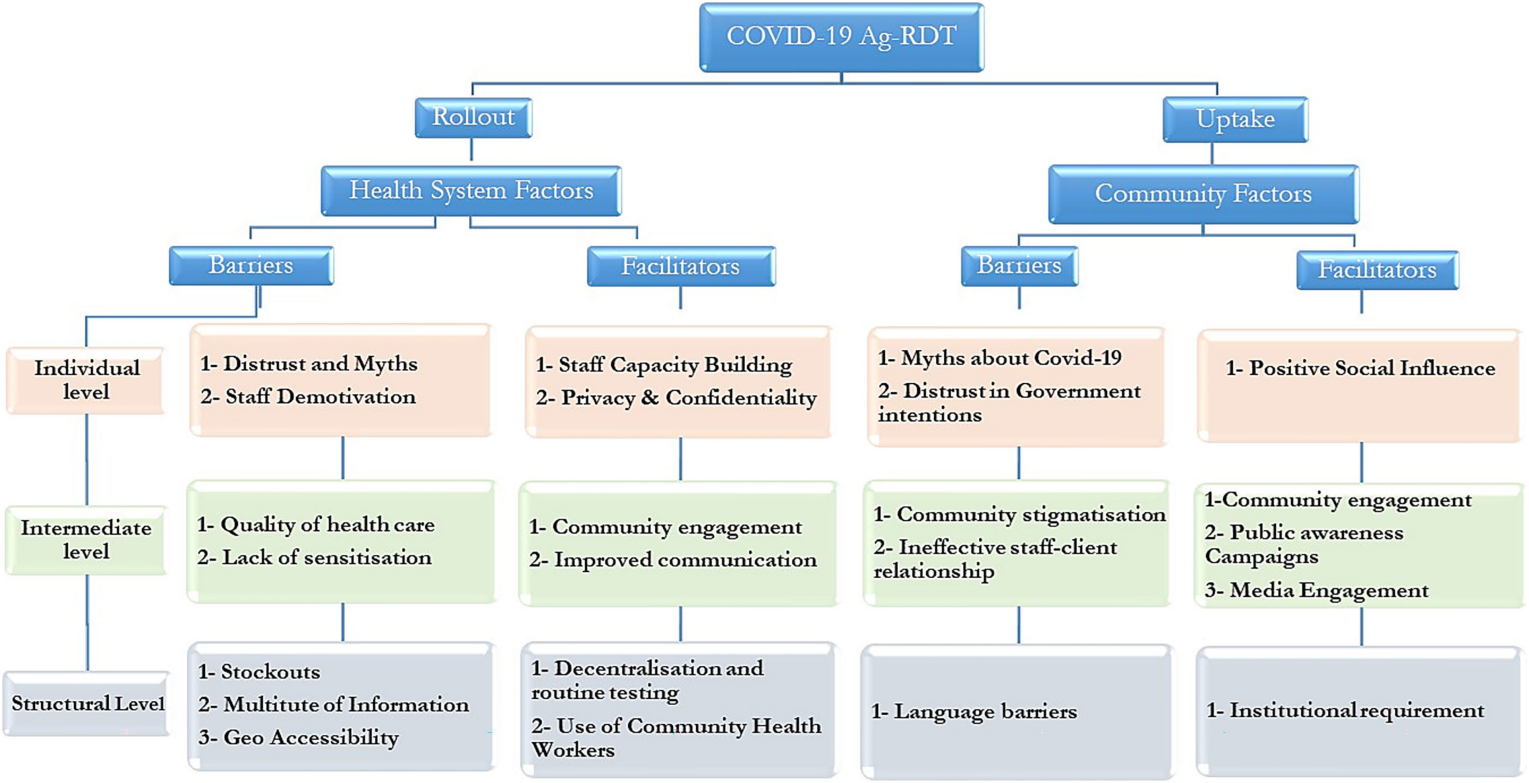
Summary of main themes for barriers and facilitators to COVID-19 Rollout and uptake.

A team of three (KEN, EESR, PTNM) conducted a manual systematic thematic analysis using an inductive coding approach to explore barriers and facilitators of COVID-19 Ag-RDT roll out and uptake. The process involved familiarization with the data, organizing segments into initial codes, and categorizing these into emergent sub-themes, which were then interpreted to identify key themes reflecting significant patterns. Each theme was classified as either a health system or community factor influencing testing rollout or uptake, further stratified into individual, intermediate, and structural levels (see Figure 1). To enhance inter coder reliability, two researchers (KEN and EESR) independently coded the transcripts, resolving discrepancies through consensus and verified by a third researcher (PTNM) to ensure accuracy. Supporting verbatim quotes were selected to exemplify each theme, providing illustrative evidence of participants’ perspectives. The analysis was initially conducted separately for each country and then harmonized, strengthening the robustness and credibility of the findings, and highlighting priority areas for targeted interventions (see Supplementary material 2).

To enhance the conceptual rigor of our analysis, the findings are situated within the Consolidated Framework for Implementation Research (CFIR), which provides a comprehensive structure for examining multilevel factors influencing implementation outcomes (20). The CFIR domains—namely, the intervention characteristics, outer setting, inner setting, characteristics of individuals, and process—align with the themes identified in our study (see summary in Table 3). Situating our findings within the CFIR framework offers a structured lens to understand the complex, interacting determinants of COVID-19 testing uptake and informs targeted strategies for effective implementation.

**Table 3:** Summary of key themes according to the CFIR.

CFIR domain	Construct	Themes from the data	Type (barrier/facilitator)
Inner setting	Available resources	Stockouts	Barrier
	Implementation climate	Staff capacity building, privacy and confidentiality	Facilitator
	Readiness for implementation	Lack of sensitization, Geo accessibility	Barrier/Facilitator
	Structural characteristics	Decentralization, Routine testing	Facilitator
Outer setting	Patient needs and resources	Distrust, Myths, Language barriers	Barrier
	Cosmopolitanism	Community engagement, Media engagement	Facilitator
	External policy and incentives	Decentralization, Use of CHWs	Facilitator
Characteristics of individuals	Knowledge and beliefs	Myths, Distrust, Community stigma	Barrier
	Self-efficacy and motivation	Staff demotivation	Barrier
	Peer influence	Positive social influence	Facilitator
Intervention characteristics	Design quality and packaging	Use of CHWs, communication strategies	Facilitator

## Results

### Sample characteristics

Thirty-six FGDs were conducted across six countries, involving 388 participants (Table 1). The majority of participants were female (52%), with the 26–35 age group being the most represented (38%) and those over 45 the least represented (14%). The results of the study are structured and presented in sections according to the main thematic areas and supported with quotes. A summary of the final themes generated are presented in Figure 1, while general codes and subthemes are included in Supplementary material 2.

Quotes will be referenced as follows;

CH = Chad, BF=Burkina Faso, CBZ = Congo Brazzaville, SL = Sierra Leone, CMR = Cameroon, NG = Niger, HW=Health Worker, CM = Community Member.

## Barriers to COVID-19 testing

### Health system barriers (individual level factors)

- Distrust and myths

Distrust and prevailing myths among health personnel significantly hindered the rollout of COVID-19 rapid diagnostic testing. Many healthcare workers expressed skepticism regarding the government’s motives, believing that COVID-19 was exaggerated for economic gain, particularly perceiving inflated positive cases as a means to secure funding from partners. This skepticism was compounded by widespread conspiracy theories and misconceptions surrounding the virus, such as the belief that it was created for political reasons;

*“Covid is an infection created by powerful Nations for economic reasons…and the government often inflates positive cases to obtain more funds from partners” (HW-CH)*.

*“Many believe that Covid-19 stems from the political war between the US and China, these ideologies slow test roll out” (HW-NG)*.

*“Using even water on some test kits gives a positive result making it difficult to willingly roll out the test to the community” (HW-CMR)*.

- Demotivation of staff

Staff demotivation emerged as a significant barrier to the effective rollout of COVID-19 testing, with many healthcare workers experiencing low morale due to factors such as low community acceptance of the tests and inadequate remuneration. This lack of motivation, particularly among community health workers, hindered their engagement in testing activities, as insufficient resources made it challenging to effectively communicate important information to the population.

*“Like myself too, I am demotivated. It's me who sends data, I pay my credit to do it, I go to the center to take the data and send, but I'm not motivated so, I no longer send the data, meanwhile there's a lot of data lying around here…” (HW-CBZ)*.

*“We the medical community are not motivated because the Ministry of Public Health gives us nothing so what advantage would I have to go raise awareness or carry out the test in the community.” (HW-SL)*.

### Health system barriers (intermediate level factors)

- Quality of Healthcare delivery and lack of sensitization

Most health personnel noted issues such as a lack of privacy and inadequate communication regarding suspected COVID-19 cases, which could often lead to stigmatization. In contrast, some health practitioners believed that many individuals were unaware of the COVID-19 testing process and require education about the disease. Without this awareness, the rollout of testing may fail to achieve its objectives and could pose risks to health personnel in certain situations.

*“To take the test, Chadians want it to be done in secret because others say that AIDS has become better since if you are positive for the COVID 19 test, the whole neighborhood is talking about you. Some also think that by taking the test they are transmitting the disease to them or giving false positive results to quarantine them…So, speaking of the test, there is still a lot to do about it when it comes to raising awareness.” (HW-CH)*.

*“Factors which have demotivated people to take the test is that information does not get to the community. The population does not often know that the test is free… (HW- SL).*

### Health system barriers (structural level factors)

- Multitude of information

The multitude of information sources from conventional media outlets to social media platforms and the numerous rumors caused health practitioners themselves to be doubtful. They had difficulties coping with the rate at which scientific knowledge for staff capacity building was provided. They stated that if they are unsure of their actions and messaging, the public will also be skeptical about taking the test. The doubts held by some medical practitioners about the disease were evident when some chose not to get tested, as explained by healthcare personnel from Congo;

*“Again, there are a lot of controversies about this COVID 19, it is hindering us the front liners, we don’t even have confidence in the whole issue. So, for us that do not even have confidence in the test how can we encourage people to get tested. So I for one, there is a whole fraud behind this thing, it’s so suspicious, let me be sincere, it is really so suspicious…” (HW-CBZ)*

*“There are so many messages. Let them come up with one particular message so that we can take it, because today, you say this, tomorrow or after 2 months, you come and change it, it’s like making a fool of us, it is really wrong…”(HW-CBZ)*

- Absence or Stock out of COVID-19 testing commodities

Most Heath personnel agreed that a significant barrier to the rollout of the COVID-19 Ag-RDT was the absence or stock outs of commodities, a challenge noted in several study countries.

“*There is low use of RDT tests by health workers during consultations due to low supply. (HW-BF)*.

*“Sometimes there is a lack of personal protective equipment to do the test because the ministry does not provide it on time” (HW-CH)*.

*“…The unavailability of the test in health centers in general and peripheral areas in particular. The community must have the COVID test nearby to allow everyone to take the test.” (HW-SL)*.

*“There is unavailability of reagents and ancillaries at the level of the testing centre” (HW-CBZ)*.

- Geographical and Financial accessibility of testing sites

As noted previously, lack of information hindered access to COVID-19 testing interventions, as beneficiaries often did not know where to get tested. Additionally, COVID-19 PCR testing sites were primarily located at reference hospitals in cities, making it difficult for citizens to travel long distances to these locations.

*“For me, before, it was difficult for a person who lives in Massengo (a remote district of northern Brazzaville) to come to Poto-Poto or come and take the test at the national laboratory, it was not easy, but with the RDTs at the integrated health center, it is close and people will come and get screened. “(HW-CBZ)*.

### Community barriers (individual level)

- Myths about COVID-19

Misinformation surrounding COVID-19, including doubts about its existence, personal beliefs and fear of vaccination significantly affected testing uptake within the community. Most community participants were of the opinion that Health practitioners themselves disseminate inaccurate information making it challenging to persuade individuals to get tested. Social media also emerged as a dominant source of information for most citizens, but it often spread negative content and conspiracy theories that fueled widespread misconceptions about the virus. These rumors led to a general underestimation of COVID-19, which further obstructed testing initiatives. As a result, low testing rates persisted, undermining public health interventions designed to control the pandemic.

*“COVID-19 doesn’t exist, if it does, it affects only Caucasians…the African Blood can kill the virus…the virus cannot Survive the hot African Climate…” (CM-CH).*

*“COVID can be treated with traditional medications or by prayers so no need to take the test… when you take the test, you are proposed the vaccine and many say that after taking the vaccine, you will turn into Chimpanzees and monkeys after some years…” (CM-CMR).*

*“There is the fear that doing the test may lead to proposal of taking the vaccine which reduces life expectancy and causes sexual weakness, even health personnel tend to say that the vaccine, causes sterility, and they themselves refuse to do the test on the pretext that it hurts” (CM-CH).*

*“…people were reluctant to take the test, because of what was being said on social media networks: if you take the vaccine you will have this or that, you even risk developing another disease and so on…Generally speaking, social media networks were detrimental for sensitization and have also played a big role in stigmatizing people…” (CM-CBZ).*

### Distrust of government intentions

Consistent with insights from healthcare providers, community participants frequently referenced conspiracy theories regarding the virus’s origin and alleged that governments inflated positive test figures to obtain international funding, which discouraged participation in screenings. Many believed that COVID-19 was a means for governments to profit from these funds.

*“Factors that have demotivated people to submit to the test is the lack of belief in this pandemic, sometimes the government's plot to benefit donations from partners.” (CM-CH)*.

*“Personally, I cannot take the test because those covid-19 Rapid tests, are always positive…” (CM-CBZ)*.

*“People are skeptical because to get tested for tetanus, meningitis you have to pay and suddenly for COVID-19 it's free, it makes people wonder if it's good…it’s like we are the guinea pigs for COVID -19 tests” BF*.

### Community barriers (intermediate level)

- Community Stigmatization

Stigmatization, isolation, and lack of discretion at health facilities created significant fear of COVID-19 testing across all study countries. Many individuals were concerned that positive results would lead to quarantine and loss of social interactions. While stigmatization was primarily observed at the community level, it also occurred in some health facilities.

*“In fact, people were afraid of quarantine. It is like being sent to hell. And people were saying if you go to the hospital, sick or not, they will always diagnose you of the disease. Some did not have the virus but were declared positive, so they preferred treating themselves at home.” (CM-CBZ)*.

*“The factors which have demotivated people from taking the test are first of all the fear of being positive and of being stigmatized by those around them” (CM-CH)*.

- Staff-client relationship

Perceived pain from the swab during COVID-19 sample collection deterred even willing individuals, especially if they felt uncomfortable or inadequately informed about the procedure. Many participants, reported significant discomfort while testing, exemplified by a case in Congo Brazzaville where a man fled the site due to anticipated pain.

*“We also had a similar case. The gentleman came to the center and agreed to take the test. So I put on my gloves, as soon as I held the swab the gentleman said I'm going to give the keys to a person who is outside I'm coming back and he was gone forever. This was related to fear of pain despite the counselling.” (HW-CBZ)*.

*“Many Staff communicate poorly to clients and do not take time to sensitize patients on the testing procedure, there is also no confidentiality, this discourages people from getting tested for COVID-19” (CM-SL)*.

### Community barriers (structural level)

- Language barrier

Health personnel and community health workers struggled to sensitize populations who only understood their local language. This linguistic challenge rendered education and sensitization efforts during the COVID-19 testing uptake inadequate.

*“Breaking language barriers is very important. For example, when you want to sensitize in the East you send the easterners that speak the same language because it will boost confidentiality amongst them” (CM-SL)*.

*“Manner of approach, it should be in a simple language of the specific group of ethnicity that will encourage the community members to come out and test.” (CM-NG)*.

## Facilitators to COVID-19 testing

### Health system facilitators (individual level)

- Capacity building/ Privacy and Confidentiality

Stakeholders recommended that healthcare providers uphold professionalism, safeguard client privacy, and prevent stigmatization, suggesting regular capacity-building training to achieve this. When the community trusts health practitioners, they are more likely to undergo COVID-19 testing. Most stakeholders emphasized the importance of empowering health practitioners with accurate information about COVID-19. Consistent messaging from health personnel and community health workers will build trust in COVID testing, ultimately increasing acceptance of testing.

*“Strategies to improve Covid-19 screening should include training of competent health personnel on testing, sensitization and pre-test counselling” (HW-CH)*.

“If you create a good working atmosphere between clients and health workers in health facilities by improving confidence and privacy, this will make the client comfortable to take the test” (HW-NG).

*“… There should be coherence in all information disseminated about COVID-19; this will enhance test roll out and uptake…” (HW-CMR).*

### Health system facilitators (intermediate level)

- Communication and community engagement

Most stakeholders agreed that while some media platforms contributed to panic and misinformation, they play a crucial role in motivating the population to get tested for COVID-19. It was highlighted that community engagement initiatives can help identify and train local leaders in effective communication and sensitization techniques. Many participants noted that Information, Education, and Communication (IEC) strategies will be effective only with involvement from the Ministry of Health and if the information shared by health practitioners and community members is harmonized, verified, and consistent.

*“In my opinion it should first start with the ministry of the Health. Since they lifted the state of emergency, the community relays who go to households to talk about Covid-19 are exposed. They can be chased with a machete or with hot water. It is up to the Congolese State, to encourage people to come and take the test… they must go on television and say that even though the state of emergency has been lifted, the disease is still there… this will encourage the population to visit health centres in case of flu to see if they have COVID-19, If the state does not do this, the work will be difficult for us (health personnel)” (HW-CBZ)*.

*“To improve the uptake of COVID-19 testing, we must first educate the population about the existence of the virus, emphasizing that it is a real disease, it’s essential to encourage the community to avoid self-marginalization and stigmatization, engaging in door-to-door sensitization, supported by Community Health Workers, and targeting community venues like churches and mosques are crucial, as not everyone has access to radio and TV. Effective strategies include sensitization, communication, and involving religious leaders and local associations to reach the community. We must also reassure the population about the sensitivity and specificity of the tests, as they are reliable and yield accurate results. (HW-CH)*.

### Health system facilitators (structural level)

- Decentralization and use of community health workers

Participants emphasized the importance of decentralizing testing centers and mobilizing the community to improve access to COVID-19 tests. They favored rapid tests due to their portability, affordability, ease of sample collection, user-friendly procedures, and quick turnaround times, which are likely to encourage more people to get tested. Accessibility of testing sites and financial affordability were identified as significant motivating factors. They identified the involvement of community health workers as essential for encouraging testing, citing that community engagement increases the effectiveness of health interventions. Contributors noted that familiar local health workers who speak the community’s language enhance understanding and adherence to testing messages.

*“I believe the popularization of rapid diagnostic tests (RDTs) is beneficial. Previously, diagnosis required PCR testing at national laboratories, which was not accessible to everyone. The introduction of antigen tests in the community is a positive initiative due to their proximity to the population. Decentralizing diagnosis has encouraged people to seek testing on their own… It's like testing for malaria. Today you can go to a pharmacy, buy and conduct the test. It's less expensive and more accessible.” (HW- CBZ)*.

*“It is important to involve community relays in Covid-19 testing but Community relays must maintain strong relationships with the community. Some individuals may initially refuse to take the Covid-19 test, but seeing a familiar face can reassure them and lead to acceptance. This is an important consideration when selecting community relays. (HW- CH)*.

### Community facilitators (individual level)

- Positive Social Influence

Social influence emerged as essential in enhancing COVID-19 test uptake within communities. The participation of respected figures, particularly religious leaders who have undergone testing, significantly altered public perceptions and encouraged individuals to get tested. Addressing common misconceptions about healing through advocacy from trusted community leaders helped overcome reluctance. Participants also added that, healthcare providers must set a precedent by openly engaging in testing, which will promote a culture of acceptance and trust regarding COVID-19 testing among the population.

*“Involving religious leaders, especially those who have been tested for Covid-19, can be very beneficial. Many people who refuse testing often say, “Jesus will heal me,” citing scriptures. If these religious leaders can help convey the message, particularly those who have already taken the test, it could significantly improve the transmission of information to the communities” (CM-CMR)*.

*“The population is not adequately informed about COVID-19 testing in our communities because healthcare providers are not setting a good example; when they avoid testing, it undermines public confidence. Therefore, the medical profession should lead by getting tested first to encourage others to follow suit” (CM-CH)*.

### Community facilitators (intermediate level)

- Community/media engagement and public awareness campaign

Community members emphasized the importance of educating and sensitizing the community to recognize COVID-19 as a genuine health issue and to provide clear information about the availability of COVID testing services in health facilities. They highlighted the roles of media, community health workers, and local leaders in this effort.

*“I think awareness-raising committees should be established to hold assemblies that feature community leaders to foster acceptance and reduce obstacles to testing. Utilize peer sensitization and involve administrative, traditional, and religious authorities. Organize interactive debates to educate communities on the importance of screening, treatment, and vaccination against COVID-19, while also combating misinformation about the disease”. (CM-NG)*.

*“Community engagement with stakeholders is crucial to support discussions between health workers and the community. If stakeholders endorse the health workers' messages, the community is more likely to accept them. Additionally, conducting radio sensitizations, health talks, and radio jingles can also help mitigate concerns.” (CM-SL)*.

### Community facilitators (structural level)

- Institutional requirements

**Figure 2:**
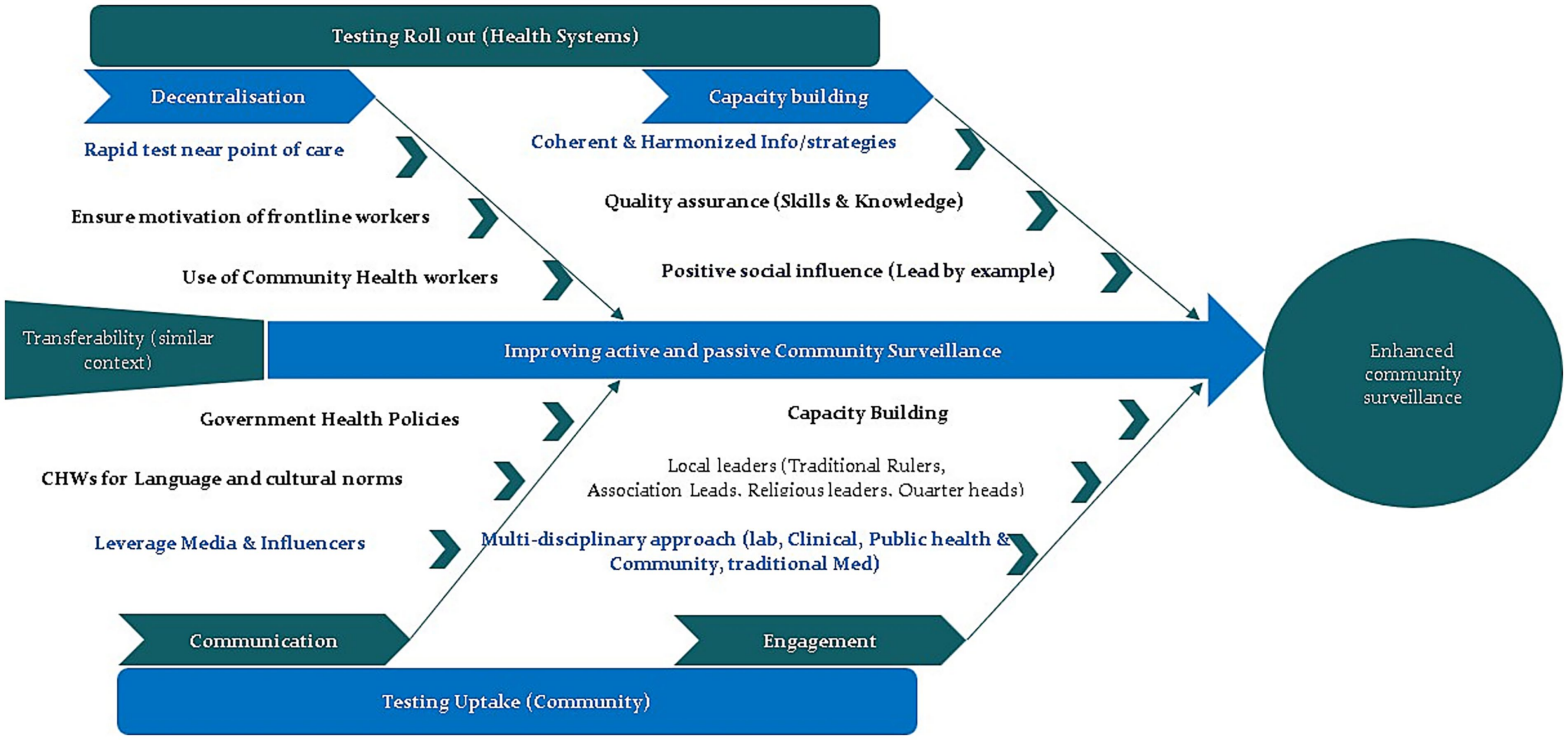
Summary of research findings (strategies to improve community testing).

Some participants identified institutional obligations as a motivating factor for COVID-19 testing, highlighting that mandatory policies by the government are generally seen as necessary for acceptance. They believe that constraints, such as requiring COVID test results to access certain services and public places, can encourage more people to get tested. Additionally, a common sentiment across countries in this study is that many individuals get tested primarily due to travel requirements, both nationally and especially internationally (see Figure 2).

*“Also there are some companies which require the covid-19 test from their staff or for recruiting… Note also the travelers who have the obligation to take the test” (CM-CH)*.

*The main motivation for people to take the COVID-19 test has been travel requirements, as crossing borders necessitates proof of a negative test. (CM-NG).*

## Discussion

This study explores multifaceted barriers and facilitators influencing the rollout and uptake of rapid diagnostic COVID-19 testing across six African nations, unveiling significant insights to optimize future health interventions.

### Health system-related factors

The findings reveal that the effectiveness of the COVID-19 Ag-RDT rollout was substantially compromised by health systems’ challenges. Health personnel’s distrust emanates from contradictory information regarding COVID-19 management from various sources, which underscores the vital necessity for harmonized communication strategies. Similar issues have been documented in other African settings, where misinformation and inconsistent messaging undermine health system responses (21). Health practitioners expressed skepticism about tests and vaccines, primarily sparked by misinformation spread through social and mainstream media. This skepticism resulted in a ripple effect, whereby doubts among healthcare providers led to diminished testing uptake within communities, exemplifying the significant roles of both trust and confidence in influencing health behaviors (22). Moreover, accessibility barriers, such as stock outs of testing commodities and geographical challenges, compounded the lack of trust in testing facilities. This aligns with prior reports emphasizing that supply chain disruptions are a persistent obstacle in resource-limited settings (15, 23).

A noteworthy aspect lies in the perceived quality of healthcare service delivery, wherein poor communication, lack of privacy, and stigmatization deterred individuals from getting tested. Improving healthcare service delivery characterized by confidentiality, respect, and active community engagement has been shown to significantly increase testing acceptance (24). Professional conduct among healthcare providers, coupled with continuous training to ensure a consistent message about COVID-19, could further assuage community fears and encourage participation; as supported by implementation research emphasizing the importance of trust (25).

### Community-related factors

The role of community perceptions around COVID-19 significantly influenced testing uptake. Prevailing conspiracy theories and misinformation, often amplified by social media, fostered mistrust in both government intentions and the health systems overall. Many individuals perceived COVID-19 as a myth or a conspiracy to exploit the population, deterring them from participating in health initiatives. This perspective aligns with previous studies documenting that community beliefs can substantially affect health behaviours, particularly in marginalized settings (21). Addressing these misconceptions through tailored community sensitization and education could create a more conducive environment for testing acceptance; an approach recommended in infectious disease control literature (26).

Moreover, the impact of language barriers was salient within the discussions. Community relay agents, who can effectively convey health information using local languages and culturally relevant approaches, appeared integral in bridging the gap between health services and the communities, hence increasing testing uptake (14). The deployment of trustworthy community members to champion COVID-19 testing could foster improved community relations and enhance overall engagement with health services as supported by prior research (26).

### Government policies

The study also highlighted the crucial effect of government health policies on testing uptakes, such as the cessation of emergency status and initiatives like mandatory testing for travelers. These policies directly shaped public behavior regarding testing, indicating that mandatory approaches tend to push higher participation rates. Similar observations have been documented where policy-driven mandates and institutional requirements significantly influence health-seeking behaviors (21). Positive insights emerged regarding decentralized testing methods, enhancing both geographical and financial accessibility. Introducing rapid diagnostic testing closer to communities not only made testing more approachable but also allowed for real-time results, minimizing wait time; a considerable factor influencing individuals’ decisions to pursue testing (6).

### Cross-country comparison of barriers and facilitators

Our qualitative exploration across six diverse African countries—Cameroon, Chad, Burkina Faso, Niger, Sierra Leone, and the Republic of Congo—revealed both common and country-specific factors influencing the rollout and uptake of COVID-19 rapid diagnostic testing. To strengthen our understanding and inform tailored interventions, we undertook a systematic comparative analysis of these findings, summarized in Table 4.

**Table 4:** Comparative summary of country-specific barriers and facilitators.

Factor	Cameroon	Chad	Burkina Faso	Niger	Sierra Leone	Congo-Brazzaville	Common themes
Misinformation and myths	Pervasive, often fueled by social media	Similar, with some conspiracy theories	Similar, with local misconceptions	Similar, especially regarding traditional beliefs	Moderate, with some myths about COVID-19 severity	Similar	Yes
Distrust in Government /conspiracies	Present, linked to economic motives	Present, linked to political motives	Present	Present	Present	Present	Yes
Supply chain issues	Noted, with shortages	Significant stock outs of test kits and PPE	Frequent shortages	Frequent shortages	Less prominent	Noted	Yes
Language barriers	Addressed via local language community relays	Significant, requiring local language communication	Critical, especially in rural areas	Critical	Major obstacle	Major obstacle	Yes
Community engagement	Religious leaders, local influencers	Religious leaders, community elders	Religious and traditional leaders	Community committees	Peer influence, community sensitization	Religious leaders	Yes
Decentralization and access	Home-based testing, community sites	Community testing centers, mobile units	Rapid tests at community health centers	Community testing, proximity	Community health workers, outreach	Community-based testing	Yes
Policy and institutional drivers	Travel requirements, institutional mandates	Travel mandates, workplace policies	Travel restrictions, institutional policies	Travel mandates	Community-driven, less formal policy influence	Travel requirements	Yes

Shared Barriers and Facilitators: Across all six countries, pervasive misinformation and myths about COVID-19—ranging from doubts about the virus’s existence to fears about vaccines—consistently impeded testing efforts. Similarly, distrust in government intentions and conspiracy theories regarding inflated case numbers for financial gain were recurrent themes. Supply chain issues, particularly stock outs of testing commodities and PPE, emerged as prominent barriers in multiple contexts, notably in Chad, Sierra Leone, and Burkina Faso.

Facilitators such as community engagement, involving trusted local leaders and religious figures, and decentralization of testing sites—bringing diagnostics closer to communities—were universally recognized strategies to enhance acceptance and access. Capacity building of health workers, maintaining confidentiality, and leveraging media campaigns also played crucial roles across settings.

Country-Specific Nuances: Despite these shared themes, distinct contextual factors shaped the implementation landscape:

Cameroon and the Republic of Congo emphasized the importance of community relays who speak local languages and foster trust, addressing linguistic and cultural barriers. In Cameroon, the introduction of home-based testing and management was particularly highlighted, reflecting localized adaptations to improve access.
Sierra Leone and Niger faced pronounced language barriers and community stigmatization, necessitating tailored communication strategies in local languages and efforts to normalize testing to reduce fears of quarantine and social exclusion.
Chad and Burkina Faso reported significant stock outs and logistical challenges, underscoring the need for strengthened supply chains tailored to their infrastructural capacities.
Travel and institutional requirements such as mandatory testing for border crossings and employment were more prominent motivators in Burkina Faso and Chad, indicating that policy-driven incentives vary across contexts.
Religious and traditional leaders played a more prominent role in Cameroon and Congo in influencing community perceptions, while in Sierra Leone, media campaigns and peer influence were more effective.
These similarities underscore the need for coordinated, high-level strategies to address common challenges like misinformation and distrust, while the differences emphasize the critical importance of context-specific approaches that consider local infrastructure, cultural dynamics, and communication needs.

### Implications for future health interventions

The insights gained from this study underscore that multifactorial approaches must be adopted to address barriers and leverage facilitators in future pandemic responses, particularly related to rapid diagnostic testing. This involves fostering collaborations between government entities, healthcare workers, and community stakeholders to ensure a cohesive response to health crises.

Future interventions should prioritize educational campaigns that counteract misinformation while emphasizing the effectiveness and safety of rapid diagnostic testing. Additionally, ensuring optimal resource allocation, increasing the presence of community health workers, and maintaining open lines of communication between health practitioners and community members can collectively enhance testing uptake.

These approaches should be tailored to local contexts, considering factors like linguistic diversity, infrastructural constraints, and cultural norms, to enhance effectiveness and trust.

## Conclusion

Comprehensive understanding of the barriers and facilitators to testing at both health system and community levels is essential for mitigating the impact of future pandemics. Engaging health practitioners and communities through informed decision-making can foster resilience and improve health outcomes. As highlighted in this study, trust, effective communication, and inclusive policymaking are pivotal in enhancing the rollout and uptake of COVID-19 testing in African nations; lessons that remain highly relevant for future public health initiatives. While broad strategies such as community engagement and decentralization are universally beneficial, their implementation must be adapted to specific cultural, infrastructural, and policy contexts to maximize impact and ensure sustainability.

### Limitations

This study employed qualitative methods, which means that the findings are closely tied to the specific participants involved and may be most applicable to similar contexts. It may be possible that some participants’ views on COVID testing may have evolved since the time of the interviews.

## Data Availability

The original contributions presented in the study are included in the article/Supplementary material, further inquiries can be directed to the corresponding author.
