# Supplementary material for: Factors influencing the rollout and uptake of COVID-19 rapid diagnostic testing: qualitative insights from six African nations
Source: Front Public Health. 2025 Oct 15;13:1551907. doi: 10.3389/fpubh.2025.1551907 (PMC12568697; doi:10.3389/fpubh.2025.1551907)
Supplement: Supplementary file 1 [file Supplementary_file_1.pdf]

Table 2. Sites supported under the project ***(Catalytic Support to Roll out/Scale up use of SARS CoV-2 AgRDTs in six African Countries)***

Country	Zone	Name of site supported	# of sites
**Sierra Leone**	Western	Hastings CHC, Laka Hospital	
	East	Jembeh	
	South	Mendewa CHC, Bo Police CHC, UMC Taiama CHC	
	North	Bumbuna CHC, Kamabai CHC, Masiaka CHC, Magburaka, Matotoka	**12**
**Niger**	Tahoua	CHR Tahoua, Cabinet Sawki Plus, CSI (Ama, Dakache, Founkoye, Garkawa, Guidan Idder, Koloma, Koweit, Laweye Goge, Maboya Amare, Malbaza, Massalata, Mounwadata, Tseranaoua, Wadata, Ketare Illela, Sabon Gari, Urbain Illela), CSME Tahoua, Hd Tahoua Ville, Koufan Tahoua	**22**
**Congo Brazzaville**	Brazzaville ARR1, 2 & 3	CSI Terinkyo, Diata, Mamba Niania, Kingouari, Bissita, 3 Martyrs/Q31, Q24, Q32	
	Brazzaville ARR 4, 5 & 6	CSI Mounkondo, Jan Vialle, Marien Ngouabi, Maman Mbouale, La Rosee, Intendance	
	Brazzaville ARR 7, 8 & 9	CSI Indzouli, Kibouende, Filgence Mbelolo, Potot Djoue, Nkombo Matari, Jacques M'pio	**21**
**Chad**	Djamena Sud	CHU BS, HNDA, Hotital de L'Union,	
	Djamena Nord	HP, HPZ	
	Djamena Centre	CHU ME, Roi Facial, CHU RN, HMI, HSCK	
	Toukra	Ngueli, Toukra	
	Djamena Est	Gazator, HATC, HR	**15**
**Burkina Faso**	Ouagadougou	**Baskey District** (CMU de Samandin, Gounghin 6 & 7, Pogbi)	
		**Bogodogo District** (CM de Saaba, CSPS de Dassasgho, CMU du Secteur 52)	
		**Boulimiogou District** (CMA de Pissy, CMU de Nagrin, CM de Tanghin Dassouri, CSPS de Pissy 17,	
		**Nongr-Massom District** (CMU de Secteur 21, CSPS Secteur 19, CSPS Secteur 13, CSPS de Bangpore,	
		**Signonghin District** (CSPS du Secteur 16, Bissighin, Pabre, Yagma,	
	Sentinel Sites	Site sentinelle de Ouagadougou, Houndé, & Bobo-Dioulasso	22
**Cameroon**	Limbe Health District	**Bota health area** (DHL & CDC Bota)	
		**Idenau health area** (CMA & Stripes)	
		**Sea Port Health Area** (CMA Limbe & PHC)	
		**Zone 2** (RHL, Larosbi Clinic)	**08**
**GRAND TOTAL**			**100**
