# Supplementary material for: Factors influencing the rollout and uptake of COVID-19 rapid diagnostic testing: qualitative insights from six African nations
Source: Front Public Health. 2025 Oct 15;13:1551907. doi: 10.3389/fpubh.2025.1551907 (PMC12568697; doi:10.3389/fpubh.2025.1551907)
Supplement: Supplementary file 2 [file Supplementary_file_2.pdf]

Country	Barriers		Facilitators	
	Ag RDT Rollout	Ag RDT Uptake	Ag RDT Roll out	Ag RDT Uptake
**Chad**	**Staff Demotivation**<br>1) low acceptance from the community<br>2) Low or no remuneration of Staff<br>4) Insufficient PPIs and other consumables<br><br>**Myths**<br>Covid is an infection created by powerful<br>Nations for economic reasons<br><br>**Skepticism about Government intentions**<br>1) Some Staff believe that the government inflates positive cases to obtain more funds from partners	**Myths**<br>1) COVID-19 doesn't exist, if it does, it affects only Caucasians<br>2) African Blood can kill the virus<br>3) The test is way to inoculate the virus in the body<br>4) All positive results are fake, it is just a way to get funding from international partners<br>5) The virus cannot Survive the hot African Climate<br><br>**Stigma related to a positive result**<br>1) By family members<br>2) By community members<br><br>**Staff related Factors**<br>1) Most health personnel refuse to take the<br>test<br>2) lack of pre-test-counselling<br><br>**Vaccine Hesitancy**<br>Fear that doing the test may lead to proposal of taking the vaccine which reduces life expectancy/sterility	**Capacity Building**<br>1) Train more staff in testing (task shifting)<br>2) Engage more CHW to improve staff capacity<br>3) Train staff on communication,<br>sensitization and pretest counselling<br><br>**Community engagement**<br>**1)** Select Community leaders and train them in communication and community sensitization<br><br>**Surveillance**<br>1) Include covid as a routine test and provide adequate testing kits<br><br>**Decentralization**<br>1) Create more testing spots for RDT to improve roll out and access<br><br>**Staff sensitization**<br>1) Staff also need to be sensitized	**Community Sensitization**<br>1) Improve sensitization and communication<br>(especially in churches and mosques)<br>2) Involve mass media (Radio, TV, Social,)<br>3) Involve Association, Religious and traditional leaders in sensitization<br>4) Staff should frequently take the test to in turn motivate the community to do same<br><br>**COVID-19 Pass**<br>1) Covid pass for travelers<br>2) Covid pass for workers<br>3) Covid pass for accessing certain public areas<br><br>**Privacy and confidentiality**<br>1) Improve Privacy of testing sites to reduce stigma
**Congo-Brazzaville**	**Low Testing uptake**<br>1) The low uptake demotivates staff from routinely rolling out the test<br><br>**Pandemic Restitution**<br>**1)** The fact government has gradually deactivated the state of emergency /strategies puts health workers in conflict with	**Poor communication**<br>The population is aware that there is no state of emergency anymore but have not been correctly informed on how they need to keep taking the test<br><br>**Perceived pain**<br>If the client is comfortable during testing procedure, he will not take the test	**Decentralization**<br>Make Covid test available at district and health center level<br><br>**Improve communication**<br>The state needs to correct the information the population has, to make them understand that deactivating the state of emergency doesn't meant the infection is over	**Community sensitization**<br>1) Proper communication with emphasis on<br>the fact that the infection is still present within communities and it is therefore important to continue testing to break the chain of transmission

	population (they understand Covid is finishing and do not comprehend why they are still requested to take a test)<br>**Too many information sources** This has casted doubts in the minds of health personnel	**Misinformation**<br>Various false information circulating on the internet about non-existence of Covid and conspiracy theories makes the population scared to take the test<br><br>**Social Media Influence**<br>A lot of controversial information circulated on social media platforms		2) Involve the media and community leaders to reach a wider population<br>3) Continue to sensitize the population on the need for constant surveillance of the infection within community to prevent another outbreak
**Sierra Leone**	**Language barrier**<br>Health personnel find it difficult to sensitize populations who understand only the local language<br><br>**Distrust and politics**<br>1) Passing wrong messages<br>2) Requesting money for access to covid related facilities slows rollout<br>3) Inadequate sensitization of health care workers<br>4) Perception that the government uses covid to find money<br><br>**Managing positive cases**<br>1) Many people deny positive test which challenges test rollout<br><br>**Stockouts**<br>**1)** Inadequate PPEs slows roll out<br>**2)** Stockout of testing kits	**Traumatizing sample collection**<br>Fear of pains with nasopharyngeal swaps<br>**Inadequate Knowledge and awareness**<br>1) People are not very knowledgeable on the seriousness and consequences of the infection<br>2) Denial about existence of the infection<br>3) People think that covid doesn't kill Africans<br><br>**Community stigmatization**<br>Fear of stigma in the community after a positive result<br><br>**Staff- client relationship**<br>1) Staff communicate poorly to clients and do not take time to sensitize patients<br>2) No confidentiality<br>3) Language issues	**Staff- client Relationship**<br>To improve RDT roll put, staff need to improve on their interaction with patients	**Community sensitization and engagement**<br>1) Populations should not be sensitized but also actively engaged in the riposte<br>2) More media engagement<br>3) Actively involve religious leaders and traditional healers<br>4) Organize Routine meetings with community stakeholders to discuss Covid trends<br><br>**Bylaws**<br>1) Implement bylaws to improve testing uptake<br>2) Use social events like football to institute mass testing and sensitization<br>3) Covid 19 test should be mandatory to all symptomatic clients
**Cameroon**	**Incoherent information**<br>1) Too many incoherent information about covid, making staff not be convinced or confident to roll out test/vaccin or sensitize the population	**Cultural/religious beliefs**<br>Covid can be treated with traditional medications or by prayers so no need to take the test<br><br>**Myths**	**Home based testing and management**<br>Clients feel more at ease with their test<br>/ management done at home and<br><br>**Coherent information**	**Community sensitization**<br>Improve communication and emphasize on importance of the test<br><br>**Community engagement**<br>Involve religious and traditional leaders in<br>community sensitization

	**Personal beliefs**<br>1) Staff believe test is meant only for travelers<br>2) Staff are not convinced that covid really exist, they believe it's a scam therefore don't think it's important to continue rolling out the test<br>**False positives**<br>Using even water on some test kits gives a positive results making it difficult to willingly roll out the test to the community	1) The vaccine causes sterility, reduces life expectancy so people fear to take the test and be proposed the vaccine<br>2) after taking the vaccine, you will turn into Chimpanzees, monkeys<br>3) Inoculation of a metal in the skin from the vaccine<br><br>**Lack of immunity**<br>People don't see the need to do the test or take the vaccine because after treatment or vaccination they will be positive again	Information given about covid should be coherent so that staff can inturn sensitize and roll out testing<br><br>**Staff motivation**<br>Improve on covid staff incentives	
**Burkina Faso**	**Incoherent information**<br>1) Contradictory information on covid slows testing rollout<br>2) Conspiracy theories about the acceptance of the infection for financial aid/political reasons<br>3) Too many vaccines gives the impression that covid is fake<br><br>**Gaps in healthcare delivery**<br>Insufficient number of testing sites	**Cultural/personal beliefs/Myths**<br>1) Covid can be treated with traditional remedies<br>2) Covid cannot be propagated due to the hot sun<br>3) Covid doesn't affect Africans<br>4) Covid vaccine causes sterility and reduces life expectancy<br>5) A magnet is inoculated in the body<br>6) Covid doesn't exist<br><br>**Lack of knowledge and awareness**<br>1) Lack of awareness on the RDT test<br>2) On available testing sites<br><br>**Sample collection**<br>1) Discomfort during sample collection<br><br>**Stigmatization and lack of confidentiality**<br>For positive cases<br><br>**Epidemiology and disease physiopathology**	**Outreach Campaigns**<br>1) Organize conferences, communication campaigns in schools etc<br>2) Improve visibility of testing sites (use gadgets, posters)<br><br>**Capacity building**<br>1) Reinforce staff training<br>2) Train additional staff and community workers on sample collection and testing<br><br>**Harmonize information**<br>1) Information on covid should be consistent and so that staff can in turn sensitize and roll out testing<br><br>**Performance based funding**<br>1) Motivate sites based on performance<br><br>**Decentralization**<br>1) Improve access to RDT s	**Community sensitization**<br>1) Tell people about possibility to self-isolate home<br>2) Address misinformation related to covid by developing key messages<br>3) Staff should show the example dy doing their own test to encourage the population to do same<br>4) Involve media/film projection/games<br><br>**Community engagement**<br>1) Involve community based organization in sensitization strategy(including religious and traditional leaders)<br>2) Organize interactive educative sessions with community in markets, religious gatherings, youth and female associations.<br><br>**Institute Sanctions**<br>1) On authors of fake news on Covid 19

		-Low mortality rate due to COVID.<br>-Asymptomatic nature the disease	2) Make RDTs available at point of care<br>**Mental Health**<br>1) Integrating mental health in covid management	**COVID PASS**<br>Compulsory covid pass for travelers and workers
**Niger**	**Gaps in healthcare delivery**<br>1) Insufficient testing sites<br>2) Inadequate access to reach remote areas<br><br>**Staff sensitization**<br><br>Staff believe it is a political war between US and China, these ideologies slow test roll out	**Distrust/Myths/misinformation**<br>1) Covid exist doesn't exist<br>2) Covid is overrated by the government, whereas it is just common cold<br>3) The test is only for those who wish to travel out of the country<br>4) Someone was vaccinated and died the following day, as such no one wants to hear about Covid anymore<br>5) The test is used by researchers to come up with something more virulent<br>**Community stigmatization**<br>1) Once people discover you are sick they avoid you in the community<br>2) Once positive one is isolated and cannot cater for the family anymore<br>**Poor staff client relationship**<br>1) Patients complain not to be well received at the testing facilities<br>**Geographical accessibility**<br>1) Insufficient screening sites<br>2) Difficult access<br>**Language barriers**<br>Hinder access to testing	**Community engagement**<br>Engage and train community health workers and other community representatives in communication, sensitization and test roll out<br><br>**Outreach campaigns**<br>1) Train more staff and organize mass testing campaigns in remote areas<br>2) Implant testing sites at strategic points with high demands like markets and borders to improve roll out<br>3) Increase the number of testing sites in general<br><br>**Staff client relationship**<br>Create a conducive environment for clients.	**COVID PASS**<br>For travelers<br><br>**Community sensitization and engagement**<br>1) Organize interactive debates<br>2) Involve religious leaders<br>3) Continuously sensitize the population<br>4) Priorities peer sensitization<br>5) Routine dialogue with communities so as assess their needs and get their opinion the most feasible strategies to improve acceptability<br>6) Organize cultural events with sketch/drama or roll plays to better sensitize the population<br>7) Priorities behavior change interventions within. communities to improve health seeking behavior<br>8) Constantly revise and update messages given to the population on Covid.
