# Supplementary material for: Factors influencing the rollout and uptake of COVID-19 rapid diagnostic testing: qualitative insights from six African nations
Source: Front Public Health. 2025 Oct 15;13:1551907. doi: 10.3389/fpubh.2025.1551907 (PMC12568697; doi:10.3389/fpubh.2025.1551907)
Supplement: Supplementary file 3 [file Supplementary_file_3.pdf]

Verbal script for FGDs with Community members

Thank you for sacrificing your time to participate in this discussion. You are invited to participate in this meeting today because you are key members of this community who may have valuable information regarding COVID-19 in the community. Today we would like to ask you about your thoughts and experiences things that will either increase or reduce COVID-19 testing uptake in your community. There are no correct or incorrect answers. We're ok if there's a question you'd rather not answer. All information you provide is confidential and will not be shared with anyone outside of the study team. Your name will never be used with this information. With your permission, we would like to record you're the discussion so that we can have them accurately. The discussion may take up to one hour.

FGD guide for community members

FGD ID: _______

Date of interview	:	Moderator	:
Location of interview	:	Note taker	:
N0 participants in FGD	:	Observer	:
Duration of discussion	:		

1. What is your general opinion about COVID-19?
   Probe: origin, transmission, prevalence, susceptibility/severity, treatment
2. What do you think about COVID-19 testing uptake in your community?
   Probes: proportion tested (good, average, poor)
3. What do you think about COVID-19 testing uptake in your community?
   Probes: proportion tested, good, poor, neutral
4. What are the things that have motivated people to take up COVID-19 testing so far?
   Probes: community factors, health system factors, others
5. What are the things that have demotivated people from taking up COVID-19 test?
   Probes:
   - Population factors (knowledge and believes, conspiracy theories, …)
   - health systems related (availability of tests, cost, distance to testing sites, population related,
6. What are the common misinformation around COVID-19 in your community?
7. For each of the barrier/misinformation discussed, what are the most practical approaches to bridge the gap and enhance testing uptake in your community?
   Probes
   - Individual level strategies (individuals in the community)
   - Community-level strategies (by community)
   - Health system-level strategies

Verbal script for FGD with health professionals

Thank you for sacrificing your time to participate in this discussion. You are invited to participate in this meeting today because you are a key health professional involved in COVID-19 activities in this community. With your experience doing COVID-19 work in the community, we think you will have valuable information to share with us regarding COVID-19 testing uptake in the community. Today we would like to ask you about your thoughts and experiences about things that will either increase or reduce COVID-19 testing uptake in your community. There are no correct or incorrect answers. We're ok if there's a question you'd rather not answer. All information you provide is confidential and will not be shared with anyone outside of the study team. Your name will never be used with this information. With your permission, we would like to record you're the discussion so that we can have them accurately. The discussion may take up to one hour.

FGD guide for Health Workers

FGD ID: _______

Date of interview	:	Moderator	:
Location of interview	:	Note taker	:
N0 participants in FGD	:	Observer	:
Duration of discussion	:		

1. What is your general opinion about COVID-19?

   Probe: origin, transmission, prevalence, susceptibility and severity, treatment
2. What do you think about COVID-19 testing uptake in your community?

   Probes: proportion tested (good, average, poor) amongst community members and health professionals
3. What are the strategies implemented to enhance COVID-19 testing uptake in your community?
4. In your opinion, what are the things that have motivated people to take up COVID-19 testing so far?

   Probes: community factors, health system factors, others (travel, institutional requirements)
5. What are the things that have demotivated people from taking up COVID-19 test?

   Probes:

   - Population factors (knowledge and believes, conspiracy theories, …)
   - health systems related (availability of tests, cost, distance to testing sites, population related)
   - Government policies (imposing testing, etc…)
6. What are the common misinformation around COVID-19 in your community?
7. For each of the barrier/misinformation discussed, what strategies would you propose to bridge the gap and enhance testing uptake in your community?

   i. **Probes**

   - Individual level strategies (individuals in the community)
   - Community-level strategies (by community)
   - Health system-level strategies

FGD transcription template

FGD /IDI ID : _______

Date of interview	:	Moderator	:
Location of interview	:	Note taker	:
N0 participants in FGD/IDI	:	Observer	:
Duration of audio record	:	Transcriber	:
Duration of transcription	:	Date of transcription	:

Moderator	Respondents/participants	Transcriber's notes
